# Comparative Genetic Analysis of Durum Wheat Landraces and Cultivars Widespread in Tunisia

**DOI:** 10.3389/fpls.2022.939609

**Published:** 2022-07-13

**Authors:** Monica Marilena Miazzi, Elyes Babay, Pasquale De Vita, Cinzia Montemurro, Ramzi Chaabane, Francesca Taranto, Giacomo Mangini

**Affiliations:** ^1^Department of Soil, Plant and Food Sciences (DiSSPA), Section Genetics and Plant Breeding, University of Bari Aldo Moro, Bari, Italy; ^2^National Gene Bank of Tunisia (BNG), Tunis, Tunisia; ^3^Agricultural Applied Biotechnology Laboratory (LR16INRAT06), Institut National de la Recherche Agronomique de Tunisie (INRAT), University of Carthage, Tunis, Tunisia; ^4^Research Centre for Cereal and Industrial Crops (CREA-CI), Foggia, Italy; ^5^Spin Off Sinagri s.r.l., University of Bari Aldo Moro, Bari, Italy; ^6^Support Unit Bari, Institute for Sustainable Plant Protection, National Research Council of Italy (IPSP-CNR), Bari, Italy; ^7^Institute of Biosciences and Bioresources, National Research Council of Italy (IBBR-CNR), Bari, Italy

**Keywords:** durum wheat, landraces, SNP diversity, genetic distances, divergent loci, spike morphology

## Abstract

The durum wheat (*Triticum turgidum* L. ssp. *durum* Desf.) landraces constitute a useful natural germplasm to increase the genetic diversity in the modern durum cultivars. The Tunisian durum germplasm constitutes 28 accessions conserved in Genebank of Tunisia, which are still unexplored. In this study, a comparative genetic analysis was performed to investigate the relationships between the Tunisian durum lines and the modern cultivars and detect divergent loci involved in breeding history. The genetic diversity analyses carried out using nine morphological descriptors and the 25K single-nucleotide polymorphism (SNP) array allowed us to distinguish two groups of Tunisian landraces and one of durum cultivars. The analysis of molecular variance and diversity indices confirmed the genetic variability among the groups. A total of 529 SNP loci were divergent between Tunisian durum landraces and modern cultivars. Candidate genes related to plant and spike architecture, including *FLOWERING LOCUS T* (*FT-B1*), zinc finger CONSTANS, and AP2/EREBPs transcription factors, were identified. In addition, divergent genes involved in grain composition and biotic stress nucleotide-binding site and leucine-reach repeats proteins and disease resistance proteins (NBS-LRR and RPM) were found, suggesting that the Tunisian durum germplasm may represent an important source of favorable alleles to be used in future durum breeding programs for developing well-adapted and resilient cultivars.

## Introduction

Durum wheat (*Triticum turgidum* L. ssp. *durum*, genome AABB, 2*n* = 4 × = 28) is the 10th most important and commonly cultivated cereal worldwide, representing 5% of total wheat production with a planting area of about 16 million hectares (International Grains Council [IGC], 2020). It is produced primarily for making pasta but also for couscous and bulgur, particularly in North Africa and the Middle East. Durum is among the first domesticated crops in the Fertile Crescent about 10,000 years ago with the development of agriculture (Feldman, 2001). With human migrations, from the Fertile Crescent it reached Africa most likely by crossing two routes, one passing through Egypt toward south to Sudan and Ethiopia, and to north to eastern Libya; the second passing through Greece toward Sicily and the coasts of Tunisia, Algeria, and Morocco (Martínez-Moreno et al., 2020). In all these areas, the genetic evolution of durum was strongly influenced by environmental conditions, leading to the development of landraces well adapted to their agroecological regions (Lopes et al., 2015). Indeed, Royo et al. (2014) observed a strong relationship between agronomic performance of durum landraces and the climate of the regions where they are widespread.

North Africa is considered one of the secondary centers of diversification of durum wheat (Boeuf, 1932; Vavilov, 1951; Lala et al., 2018), where it is still a staple crop with outstanding socioeconomic value for local populations (Daaloul et al., 1997; Deghaïs et al., 2007). According to Boeuf (1932), Ducellier (1930), and Émile (1950), Tunisian durum wheat landraces constitute a remarkable natural collection of this species and the multitude of existing forms would be due to hybridizations and crosses that occur spontaneously with the varieties introduced during the Arab invasion. Studies on genetic diversity have shown that Tunisian landraces, while sharing an allelic pool with those from the Mediterranean basin, accumulated also distinct mutations over time (Kabbaj et al., 2017). However, the durum wheat germplasm of Tunisian landraces is constituted by a few recognized accessions conserved in National Genebank, most of which are still unexplored (Robbana et al., 2019). By the early 1970s, after the Green Revolution, durum landraces were progressively abandoned and replaced by improved genetically uniform modern cultivars (Bonjean and Angus, 2001; Ortiz et al., 2007). Nowadays, the durum landraces are mainly grown by smallholder farmers under low-input traditional agrosystems in the Tunisian marginal areas (Slim et al., 2019). In Tunisia, the most well-known durum landraces are Biskri, Bidi, Mahmoudi, and Jenah Khotifa. The landrace Biskri and some selection lines within Bidi were introduced in Tunisia from Algeria. Mahmoudi is considered a local landrace population with various reported origins and is known for its resistance to drought (Othmani et al., 2019). Jenah Khotifa shows purplish black glumes, black awns, and high carotenoid content (Deghaïs et al., 2007; Robbana et al., 2019).

The Tunisian breeding programs were started in the early 20th century, from French colonists who rounded up the existing landraces, evaluated their production potential, and selected the genotypes with a higher grain yield. These breeding programs resulted in the release of the pure selections of Bidi, Mahmoudi, and Jenah Khotifa. Starting from the 1970s, genetic materials obtained from CIMMYT and ICARDA led to the registration of new high-yielding semi-dwarf cultivars more suitable to intensive production systems (Martínez-Moreno et al., 2020).

A high diversity of Tunisian durum accessions has been observed using morphological descriptors according to the Union for Protection of New Varieties of Plants (UPOV) and International Board for Plant Genetic Resources Institute (Ayed and Slim-Amara, 2009; Ayed et al., 2010; Slim et al., 2011; Ouaja et al., 2021), and biochemical markers (Babay et al., 2015). The genetic diversity of Tunisian durum germplasm was also investigated using different molecular markers such as AFLP and SSR markers, which allowed us to study the genetic variation among and within Tunisian landraces and modern cultivars (Medini et al., 2005; Slim et al., 2019; Ouaja et al., 2021).

The availability of single-nucleotide polymorphism (SNP) markers generated by next-generation sequencing-based approaches (Sansaloni et al., 2011; Poland et al., 2012; Wang et al., 2014), and the release of the durum wheat reference genome, allows us to detect useful alleles to be used in the new durum breeding programs, as well as understand the genetic structure and consequences of evolution and selection history (Maccaferri et al., 2019). Several studies have investigated the genetic diversity in durum collections, including cultivars and landraces, using SNP markers (Kabbaj et al., 2017; Mangini et al., 2018; Marzario et al., 2018; N'Diaye et al., 2018; De Vita and Taranto, 2019; Robbana et al., 2019, 2021; Taranto et al., 2020, 2021; Ganugi et al., 2021). However, genetic diversity is affected by many factors that promote genetic differentiation, such as gene flow, genetic drift, mutation rates, and levels of recombination. These stochastic effects might be accentuated by the impact of natural selection and artificial selective breeding that are the main driving forces shaping genetic variation across modern cultivars and their wild and domesticated relatives. The study of molecular signatures of divergence was conducted using a large panel of durum wheat, with the aim to explore the underexplored genetic variability of landraces and wild progenitors and detect beneficial alleles to introduce in modern cultivars (Mazzucotelli et al., 2020; Taranto et al., 2020, 2022; Roncallo et al., 2021; Negisho et al., 2022). In addition to divergent selection, genes involved in convergent selection, due to independent domestication of related species (i.e., cereals), also provide promising insight into the detection of the trait related to domestication and adaptation and may benefit efforts to improve wheat breeding programs (Woodhouse and Hufford, 2019).

Considering this in mind, we aimed to (1) perform a comparative genetic analysis between landraces and cultivars widespread in Tunisia using SNP markers, (2) detect divergent and convergent loci among the groups highlighted by phylogenetic analysis, and (3) suggest putative candidate genes that are involved in the Tunisian breeding history.

## Materials and Methods

### Plant Material

A collection of 43 durum wheat accessions, including the most representative 28 lines selected within Tunisian landrace populations, nine Tunisian cultivars derived from crossing, and six international cultivars (Simeto, Mexicali-75, Creso, Iride, Polesine, Lyodl), were analyzed. The seeds were provided by the National Genebank of Tunisia, the Research Centre for Cereal and Industrial Crops, Foggia, Institute of Biosciences and Bioresources, National Research Council, and Gene Bank of Prague (Czechia) (Supplementary Table S1).

### Morphological Traits Characterization

In accordance with the guidelines by UPOV (1988) and the International Board for Plant Genetic Resources (1985), a panel of nine morphological descriptors were used to phenotype the durum accessions (Supplementary Table S1). A durum field trial with plots consisting of 1-m rows, 60 cm apart, with 20 germinating seeds per plot was used. Data were scored on 10 plants per plot, at harvesting time, on a random sampling of 15 representative spikes (split into three replicates consisting of five spikes) and were was carried out from each landrace/cultivar. The data were used to obtain a morphological clustering of accessions using the neighbor-joining clustering method using the DARWIN software v.6.0.021 (Perrier and Jacquemoud-Collet, 2006), with bootstrapping of 1,000 replicates to determine the support for each node.

### Genotyping and SNP Quality Control

The durum wheat accessions were genotyped using the Illumina wheat 25K Infinium iSelect array (https://www.traitgenetics.com) (TraitGenetics Gmb, 2022). SNP marker positions were ordered according to the physical map of durum wheat genome cv. Svevo (Maccaferri et al., 2019) available at https://www.interomics.eu/durum-wheat-genome (Interomics, 2022). The SNP data were filtered using the TASSEL software (Bradbury et al., 2007), discarding SNPs with a call rate >10% and minor allele frequency less of 1%. Finally, the data set was pruned for linkage disequilibrium (LD) (*r*^2^ = 0.50) using the SNP and Variation Suite (SVS, 2022) software v.8.4.0 (http://www.goldenhelix.com).

### Genetic Diversity Analysis

The SVS software was used to calculate the identity by state (IBS) distances among accessions and identify cases of synonymy (IBS > 0.99). A phylogenetic tree was carried out using the maximum likelihood method based on the Tamura–Nei model (Tamura and Nei, 1993) with 1,000 replicates to generate the bootstrap values in MEGA X (Kumar et al., 2016). In addition, principal coordinate analysis (PCoA) was performed to estimate the genetic diversity among durum wheat accessions. The analysis of molecular variance (AMOVA) was performed to check the significance of the variance between groups obtained from phylogenetic analysis. PCoA, AMOVA, as well as the genetic indexes, such as number of effectives alleles (*Ne*), Shannon's information index (*I*), heterozygosity observed (*h*), percentage of polymorphic loci (*PPL*), and private alleles, were estimated using the Genalex v.6.5 software (Peakall and Smouse, 2012). Additionally, gene flow (*Nm*) among populations was calculated based on *F*_*ST*_ according to Negisho et al. (2021).

### Identification of Divergent Loci

The *F*_*ST*_ at single SNP loci was estimated according to Taranto et al. (2020) by pairwise comparison between the groups identified by the phylogenetic analysis. Venn diagram was used to visualize the divergent loci (*F*_*ST*_ > 0.25) using the Venny software (Oliveros, 2007). Putative candidate genes flanking the regions of divergent SNPs were identified according to the average LD decay distance estimated in durum collection using *r*^2^ statistic at threshold = 0.20.

## Results

### Morphological Traits Diversity

**Figure 1:**
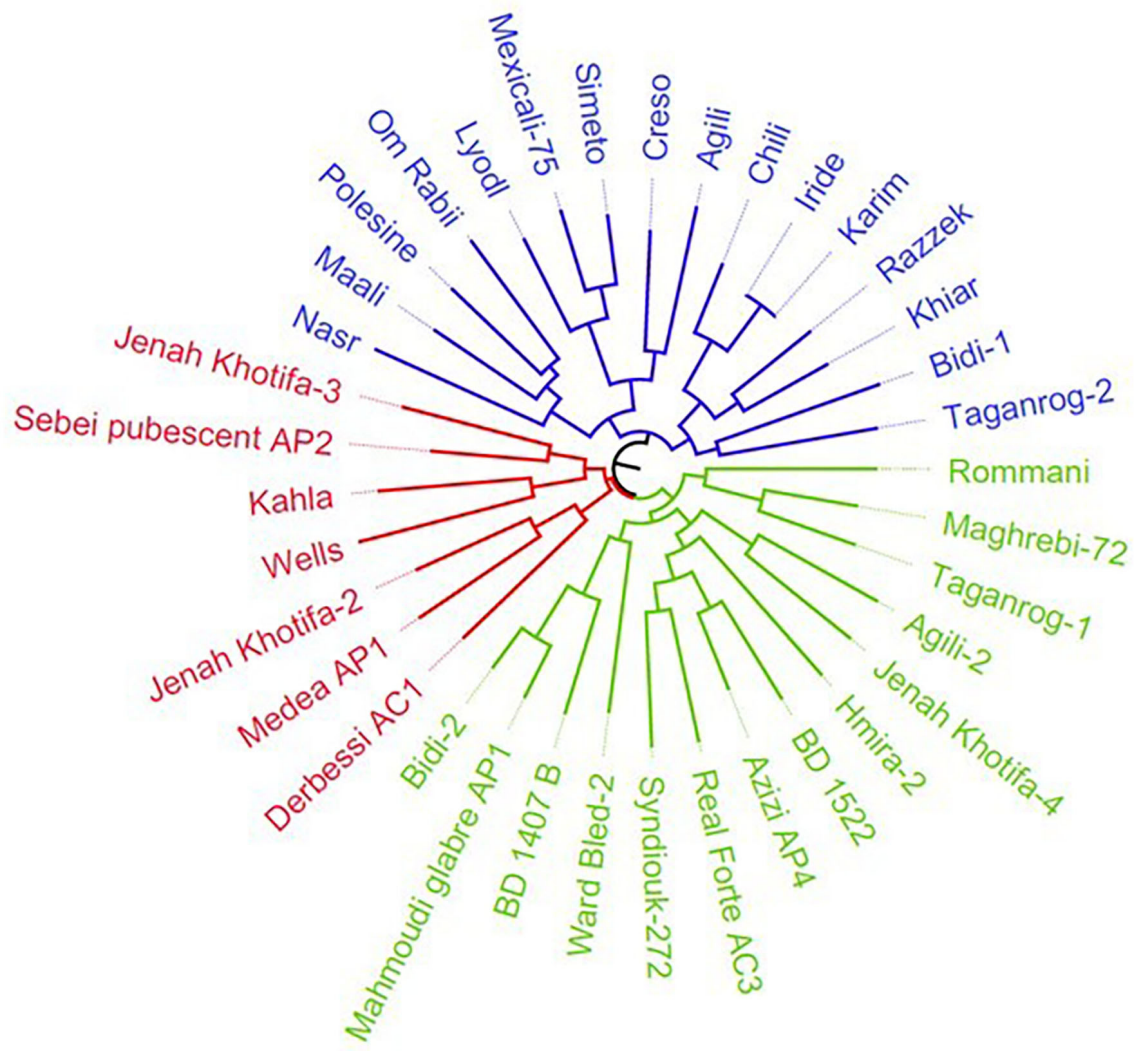
Neighbor-joining dendrogram of durum collection based on nine morphological descriptors. The Tunisian and international durum cultivars are colored in blue, while the Tunisian durum landraces group is indicated in red and green, respectively. The tree excluded the accessions identified as synonymous (Abdelkader, Mahmoudi-552, D-58-25-A, Mahmoudi, Souri Rp5, and Amel-72).

The durum wheat collection was characterized by nine morphological descriptors related to spike and kernels (Supplementary Table S1). These traits were used to generate a tree that displayed three main clusters (Figure 1). The first cluster included 12 cultivars and four lines selected within the landraces Taganrog, Agili, Chili, and Bidi, while the second and third grouped 14 and seven genotypes selected within Tunisian durum germplasm, respectively. The clustering distinguished the accessions based on spike density and glume color. Cluster I comprised mainly cultivars with higher spike density (value 7) than clusters II (value 5) and III (value 3). The accessions were also divided based on glume color; clusters I–III included the majority of accessions with white color of glume (1), reddish (2), and purple (3), respectively (Supplementary Table S1).

### Genetic Relationships and SNP Diversity

The genetic diversity of the collection was investigated by a high-throughput genotyping system based on a 25k SNP wheat array. A total of 11,746 high-quality SNPs were retained after filtering and mapped on the 14 chromosomes of the durum genome (Maccaferri et al., 2019). Filtered VCF file was submitted to the Mendeley Data repository (https://data.mendeley.com/datasets/9hnjw77mf2/2) under the doi: 10.17632/9hnjw77mf2.2. LD pruning was applied to remove markers in strong LD, thus retaining 5,555 SNPs that were used in downstream analyses.

**Figure 2:**
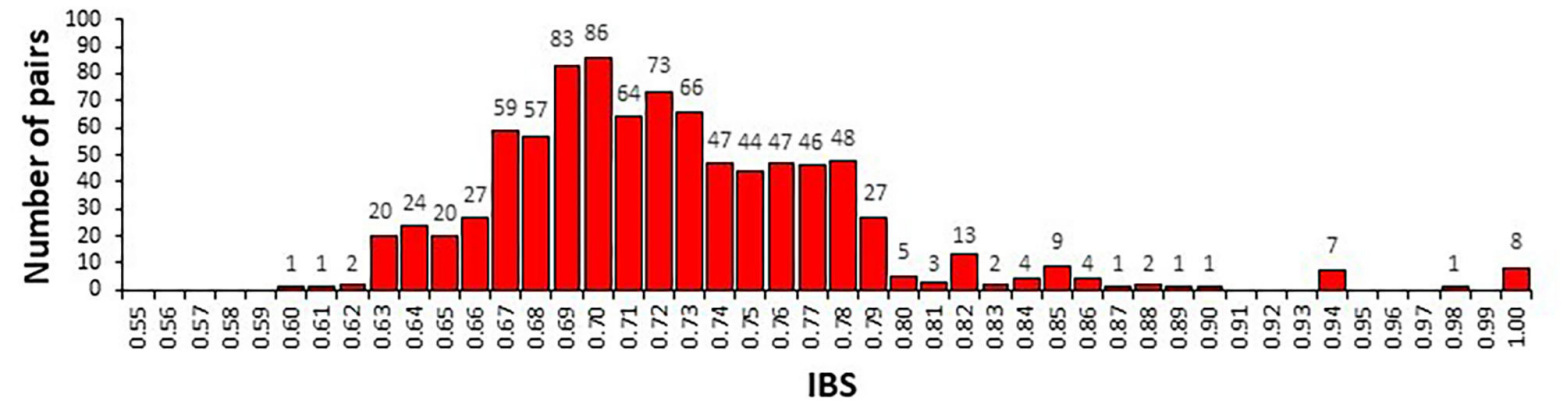
Distribution of identity-by-state (IBS) allele sharing values among 43 durum wheat samples determined by the analysis of 5,555 SNP markers.

IBS values ranged from 0.60 to 1.00 (Figure 2). Eight pairs of IBS values were higher than 0.99, suggesting the presence of synonymous accessions in the Tunisian landraces. In detail, the accessions Chili, Abdelkader, and Mahmoudi-552, as well as Mahmoudi, D-58-25-A, and Bidi-2 were synonymous. In addition, Wells and Mahmoudi glabre AP1 accessions were similar to Amel-72 and Souri Rp5, respectively. For this reason, six accessions (Abdelkader, Mahmoudi-552, D-58-25-A, Mahmoudi, Souri Rp5, and Amel-72) were discarded, obtaining a set of 37 durum samples.

**Figure 3:**
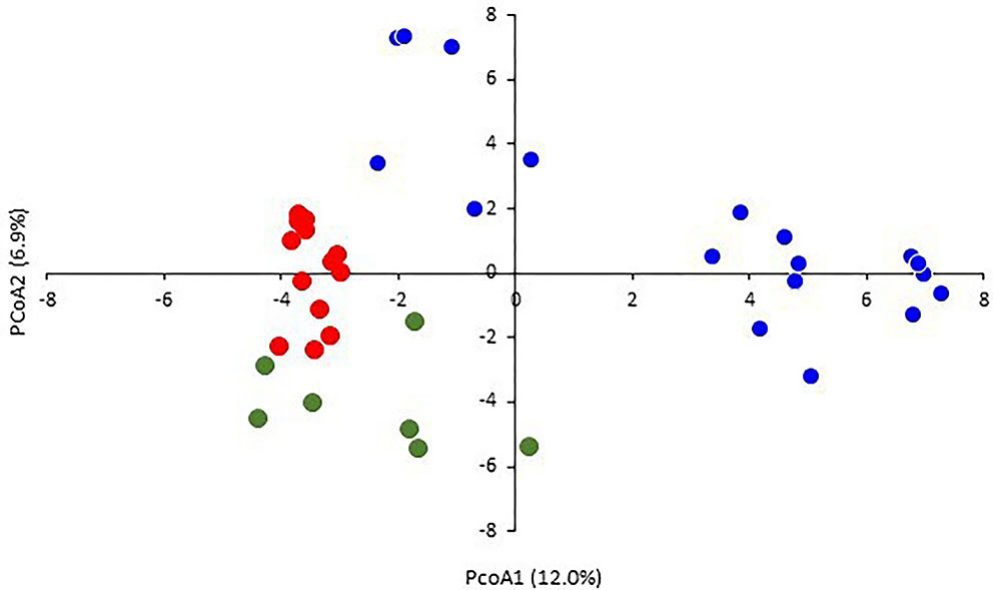
Principal coordinate analysis (PCoA) based on the genetic distances for durum wheats using 5,555 SNPs. The samples are colored in blue (modern durum cultivars, MDC), red (Tunisian durum landraces group 1, TDL-1), and green (Tunisian durum landraces group 2, TDL-2).

**Figure 4:**
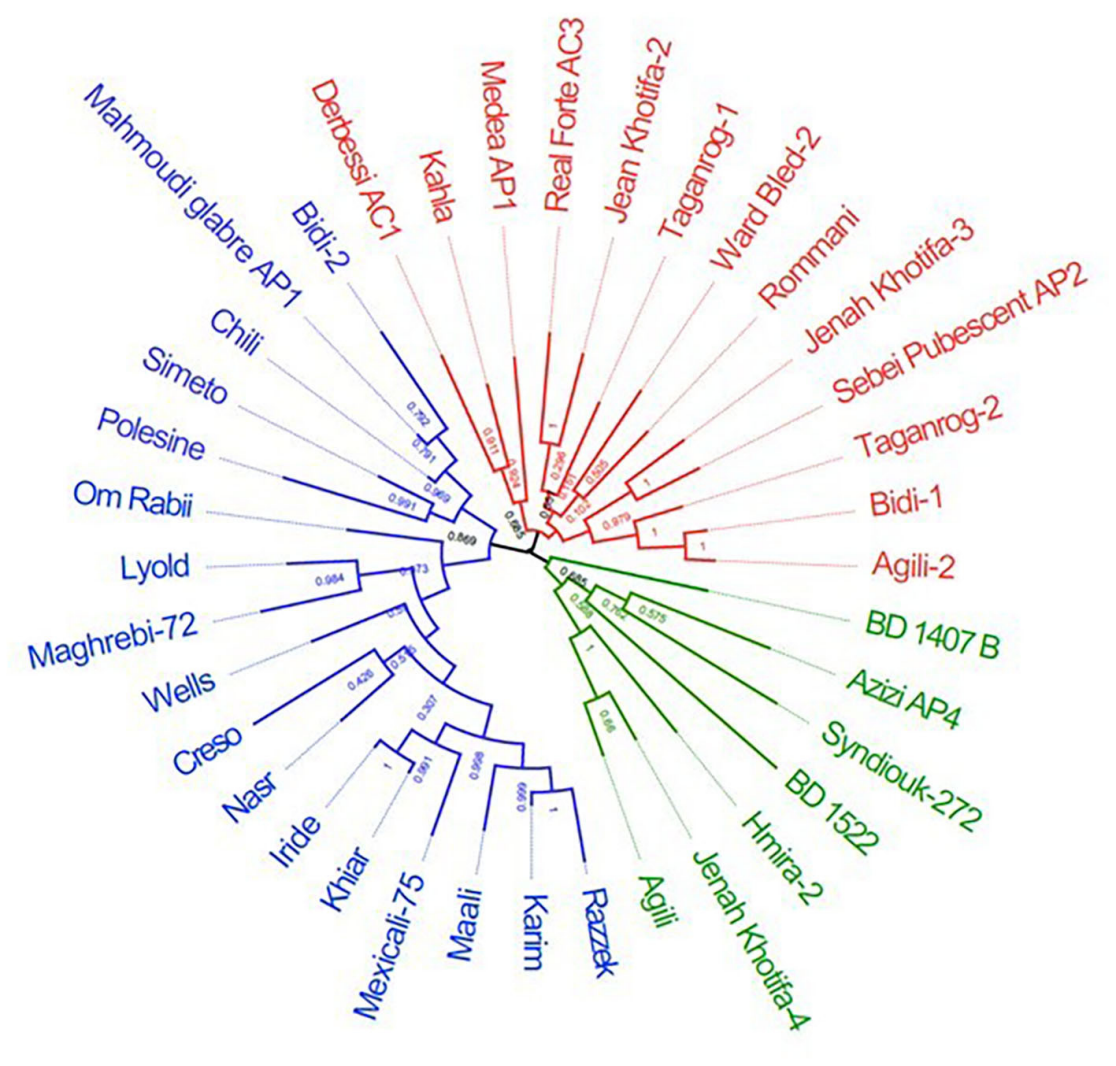
Unrooted tree constructed using maximum likelihood method based on the genetic distances for durum wheats using 5,555 SNPs. The samples are colored in blue (modern durum cultivars, MDC), red (Tunisian durum landraces group 1, TDL-1), and green (Tunisian durum landraces group 2, TDL-2).

The two-dimensional PCoA plot revealed a clear genetic diversity within the durum accessions, explaining 18.9% of the total variation (Figure 3). The accessions were distributed into four quadrants. The Tunisian landraces were included in the third and fourth quadrants while the modern cultivars were spread mainly in the first and second ones. The phylogenetic tree was consistent with PCoA, clustering the accessions into three main groups (Figure 4). The first one, modern durum cultivars (MDC), consists of 17 accessions, including the Tunisian cultivars, the international cultivars, and the three lines selected within well-known old landraces Mahmoudi (Mahmoudi glabre AP1), Bidi (Bidi-2), and Chili, respectively.

The second cluster, Tunisian durum landraces group 1 (TDL-1), included 13 lines derived from Tunisian durum landraces such as Medea (Medea AP1), Bidi (Bidi-1), Agili (Agili-2), Realforte (Real Forte AC3), Taganrog (Taganrog-1 and Taganrog-2), Jenah Khotifa (Jenah Khotifa-2 and Jenah Khotifa-3), and Romnani. The third cluster, Tunisian durum landraces group 2 (TDL-2), consists of seven accessions, including two Tunisian old varieties (BD1407 B and BD 1522) provided by the Gene Bank of Prague (Czechia), and selection lines derived from Tunisian durum landraces Azizi (Azizi AP4), Hmira (Hmira-2), and Jenah Khotifa (Jenah Khotifa-4).

The genetic fixation index (*F*_*ST*_) and gene flow (*Nm*) calculated between the three groups showed values of 0.05 and 4.7 (TDL-1 vs. TDL-2), 0.07 and 3.3 (MDC vs. TDL-1), and 0.12 and 1.8 (MDC vs. TDL-2). The AMOVA, calculated based on the three groups identified by the SNP cluster analysis, revealed that 16% of the total variation was among groups while the rest (84%) was within groups (Table 1).

**Table 1:** Results of AMOVA using 5,555 SNP markers and 37 durum wheats.

**Source of variation**	**Degree of freedom**	**Sum of squares**	**Variance components**	**Percentage of variation**	***p* values**
Among groups	2	4836.018	2418.009	16%	<0.001
Within groups	34	25964.468	763.661	84%	
Total	36	30800.486		100%	

The genetic diversity was estimated among the three clusters obtained by phylogenetic analysis. The MDC group showed the lowest number of effective alleles per locus (*Ne*) (1.38), Shannon's information index (*I*) (0.36), diversity index (*h*) (0.23), and the higher number of private alleles (552). The TDL-2 group showed the highest values for all indices, *Ne* (1.51), *I* (0.44), *h* (0.29), polymorphism (*PPL*) level (76.4%), and 421 private alleles. The TDL-1 group showed intermediate values for the indices, with a *PPL* of 71% and 331 private alleles (Table 2). The three groups showed a wide variation in loci carrying private alleles among the wheat chromosomes. The MDC group showed a high number of private alleles on chromosomes 1B, 2B, 4B, 5A, 6B, 7A, and 7B; TDL-1 group on 6A, and TDL-2 group on chromosomes 1A and 5B (Supplementary Figure S1).

**Table 2:** Number of effectives alleles (*Ne*), Shannon's information index (*I*), heterozygosity observed (*h*), percentage of polymorphic loci (*PPL*), and private alleles in durum collection and in the three groups (MDC, TDL-1, and TDL-2) defined by phylogenetic analysis.

**Group**	**Accessions**	***Ne***	***I***	***h***	***PPL* (%)**	**Private alleles**
MDC	17	1.38	0.36	0.23	73.7	552
TDL-1	13	1.40	0.36	0.24	71.0	331
TDL-2	7	1.51	0.44	0.29	76.4	421

*MDC, modern durum cultivars; TDL-1, Tunisian durum landraces group 1; TDL-2, Tunisian durum landraces group*.

### Divergent Loci Analysis and Candidate Genes

**Figure 5:**
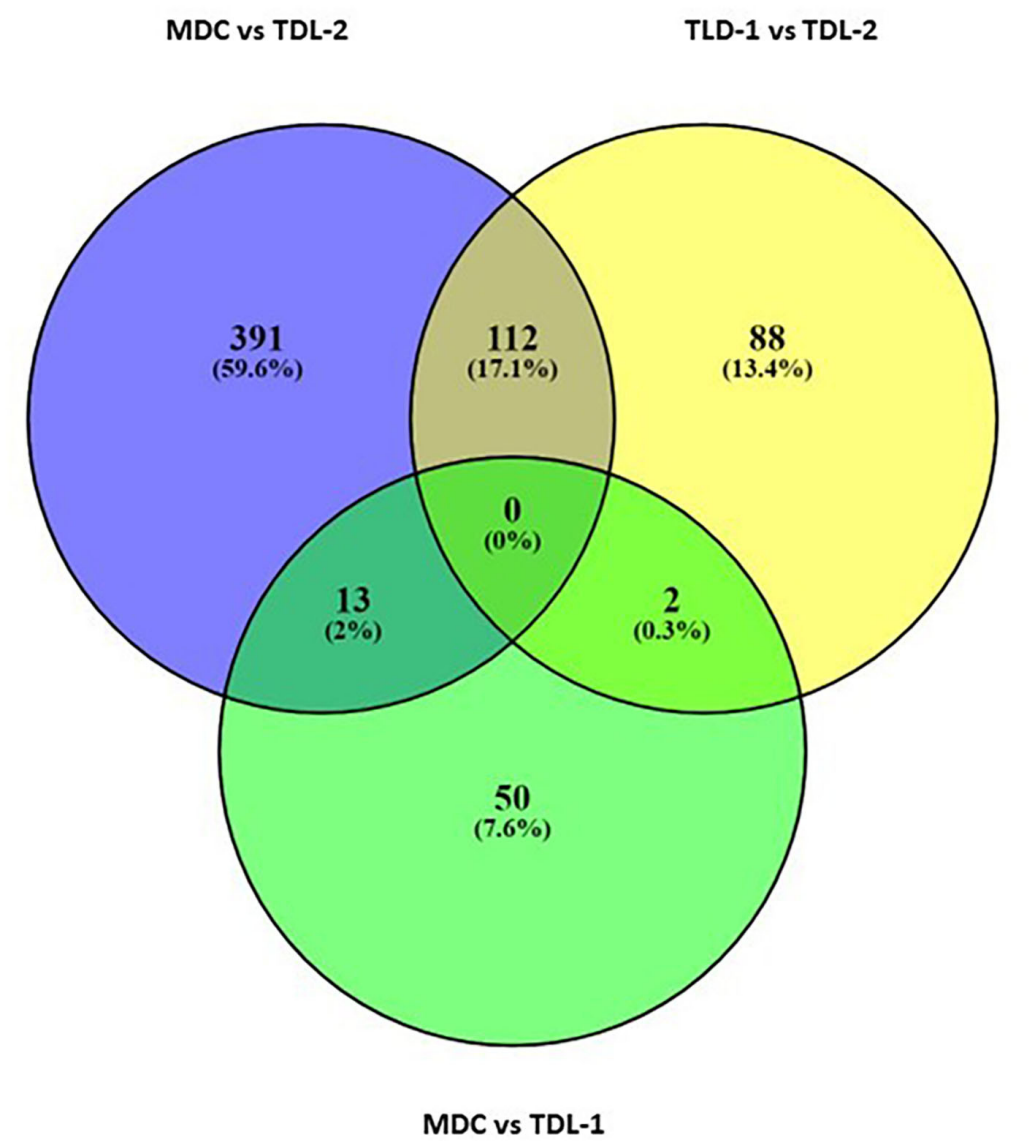
Venn diagram of divergent SNPs (with an *F*_*ST*_ threshold to >0.25) among the three populations defined with cluster analysis. The numbers in parentheses show percentages of divergent SNPs out of the total number. MDC, TDL-1, and TDL-2 indicate modern durum cultivars, Tunisian durum landraces groups 1 and group 2, respectively.

To detect divergent genomic regions among the three groups (MDC, TDL-1, and TDL-2), the pairwise fixation index (*F*_*ST*_) at individual SNP loci was calculated. A total of 529 divergent SNP loci (*F*_*ST*_ > 0.25) were found, of which 391, 88, and 50 were found in MDC vs. TDL-2, TDL-1 vs. TDL-2, and MDC vs. TDL-1, respectively (Figure 5).

A total of 322 divergent SNP loci with a *F*_*ST*_> 0.6 comparing MDC vs. TDL-2 (228), TDL-1 vs. TDL-2 (86), and MDC vs. TDL-1 (8), respectively, were found (Supplementary Table S2). To support the identification of divergent loci, candidate genes were searched using the functional annotation of durum genome cv. Svevo. According to average LD decay estimated in the durum collection using a threshold of *r*^2^ = 0.2, an interval of ±3.5 Mb around each divergent SNP was considered (Supplementary Figure S2). The core of candidate genes identified was divided into three main categories, namely, plant architecture, grain quality, and disease resistance.

Genes related to plant and spike architecture, as well as flowering time and belonging to *FLOWERING LOCUS T (FT)*, CONSTANS like, nitrogen assimilation control (*NAC*), APETALA2/ethylene-responsive factor (*AP2/ERF*), GRAS transcription factor, purple acid phosphatase (*PAP*), and nitrogen transporter gene families, were recognized (Table 3, Supplementary Table S3). *FT* genes were detected on chromosomes 2A, 2B, 3A, 5A, and 7B (Table 3). CONSTANS proteins and *AP2/ERF* were also identified. A total of 18 NAC proteins, localized on 2, 3, 5, and 7 homoeologous chromosome groups were discovered. Genes related to grain quality were also identified. In addition, several *LATE EMBRYOGENESIS ABUNDANT* (*LEA*) genes were found on chromosome 2A, 2B, 3A, 4B, 5B, 6A, 6B, 7A, and 7B (Supplementary Table S3).

**Table 3:** Core set of candidate genes detected using divergent SNP loci between the modern durum cultivars (MDC), Tunisian durum landraces group 1 (TDL-1) and group 2 (TDL-2) using *F*_*ST*_ > 0.60.

**Divergent SNP marker**	**Chr**.	**Transcript ID**	**Position (bp)**	**Gene or functional annotation**	**Pairwise comparison**
RAC875_c79370_378	1A	TRITD1Av1G018240	38,286,635	Starch composition	MDC vs. TDL-2
AX-158605815	1A	TRITD1Av1G178990	479,480,350	CONSTANS protein	MDC vs. TDL-2
AX-158540295	1B	TRITD1Bv1G001870	4,313,476	Gliadines	MDC vs. TDL-2
Kukri_c8390_1102	1B	TRITD1Bv1G002140	4,905,408	Powdery mildew resistance protein (Pm3)	MDC vs. TDL-2
Kukri_c8390_1102	1B	TRITD1Bv1G002670	6,505,318	Powdery mildew resistance protein (Pm3)	MDC vs. TDL-2
AX-94679104	2A	TRITD2Av1G010590	18,659,192	*FLOWERING LOCUS T (FT)*	MDC vs. TDL-2
AX-94446663	2A	TRITD2Av1G213030	587,743,831	CONSTANS protein	MDC vs. TDL-2
Tdurum_contig30451_88	2A	TRITD2Av1G215850	596,101,246	*APETALA2/ETHYLENE RESPONSIVE FACTOR (AP2/ERF)*	MDC vs. TDL-2
AX-94461119	2A	TRITD2Av1G266170	717,396,105	*FLOWERING LOCUS T (FT)*	MDC vs. TDL-2
AX-94674675	2A	TRITD2Av1G273210	731,322,415	*APETALA2/ETHYLENE RESPONSIVE FACTOR (AP2/ERF)*	MDC vs. TDL-2, TDL-1 vs. TDL-2
AX-95093101	2A	TRITD2Av1G273610	731,960,627	*APETALA2/ETHYLENE RESPONSIVE FACTOR (AP2/ERF)*	MDC vs. TDL-2
AX-94748705	2B	TRITD2Bv1G013720	28,067,238	*FLOWERING LOCUS T (FT)*	MDC vs. TDL-2
AX-94417710	2B	TRITD2Bv1G172950	511,050,164	*FLOWERING LOCUS T (FT)*	MDC vs. TDL-2
AX-95195012	3A	TRITD3Av1G009580	18,357,241	*APETALA2/ETHYLENE RESPONSIVE FACTOR (AP2/ERF)*	MDC vs. TDL-2
BS00110564_51	3A	TRITD3Av1G253390	680,896,807	*FLOWERING LOCUS T (FT)*	MDC vs. TDL-2
AX-94540165	4A	TRITD4Av1G254860	712,434,403	Gliadines	MDC vs. TDL-2
Excalibur_c1208_72	5A	TRITD5Av1G172070	468,247,346	*FLOWERING LOCUS T (FT)*	MDC vs. TDL-2
BS00033612_51	5B	TRITD5Bv1G013720	37,226,691	Carotenoid pathway	MDC vs. TDL-2
BS00079321_51	5B	TRITD5Bv1G205010	582,209,141	*APETALA2/ETHYLENE RESPONSIVE FACTOR (AP2/ERF)*	MDC vs. TDL-2
BobWhite_c18550_159	6A	TRITD6Av1G037420	87,195,422	CONSTANS protein	MDC vs. TDL-2
wsnp_Ex_c9502_15748469	6A	TRITD6Av1G189220	536,614,876	Starch composition	TDL-1 vs. TDL-2, MCD vs. TDL-2
BS00078124_51	6A	TRITD6Av1G225060	611,124,580	Disease Resistance Proteins RPM1	MDC vs. TDL-1
BS00078124_51	6A	TRITD6Av1G225070	611,127,679	Nucleotide-binding site–leucine-rich repeat (NBS- LRR)	MDC vs. TDL-1
BS00078124_51	6A	TRITD6Av1G225140	611,279,044	Disease Resistance Proteins RPP13	MDC vs. TDL-1
BS00078124_51	6A	TRITD6Av1G225410	612,003,088	Disease Resistance Proteins	MDC vs. TDL-1
BS00078124_51	6A	TRITD6Av1G225660	612,423,374	Nucleotide-binding site–leucine-rich repeat (NBS- LRR)	MDC vs. TDL-1
wsnp_Ex_c64847_63484965	6B	TRITD6Bv1G050550	142,276,394	CONSTANS protein	MDC vs. TDL-2
BS00082268_51	7A	TRITD7Av1G008200	13,903,571	Gliadines	MDC vs. TDL-2
BS00082268_51	7A	TRITD7Av1G008680	14,634,886	Gliadines	MDC vs. TDL-2
wsnp_Ex_c38326_45883440	7A	TRITD7Av1G226310	606,239,534	Carotenoid pathway	MDC vs. TDL-2, TDL-1 vs. TDL-2
wsnp_Ex_c38326_45883440	7A	TRITD7Av1G227500	608,633,211	CONSTANS protein	MDC vs. TDL-2
AX-94432756	7B	TRITD7Bv1G003900	9,126,441	*FLOWERING LOCUS T (FT)*	MDC vs. TDL-2
wsnp_Ex_rep_c107796_ 91279476	7B	TRITD7Bv1G038900	107,291,251	Starch composition	MDC vs. TDL-2
Tdurum_contig93081_162	7B	TRITD7Bv1G172240	544,960,925	Carotenoid pathway	MDC vs. TDL-2
AX-158592916	7B	TRITD7Bv1G224220	693,666,535	Powdery mildew resistance protein (Pm3-like)	MDC vs. TDL-2

*Divergent SNP markers, chromosome location (Chr.), transcript ID, physical position on cv. Svevo (bp), gene or functional annotations, and pairwise comparison are reported*.

Genes for starch composition were found on chromosomes 1A and 7B in MDC vs. TDL-2, while on chromosome 6A both in MDC vs. TDL-1 and TDL-1 vs. TDL-2 (Table 3). Genes involved in carotenoid pathway were also detected on chromosomes 5B and 7B in MDC vs. TDL-2, while on chromosome 7A both in MDC vs. TDL-1 and TDL-1 vs. TDL-2. Gliadin genes were divergent on chromosomes 1B, 4A, and 7A between MDC and TDL-2, while on chromosome 7A other two gliadin genes were identified in TDL-1 vs. TDL-2. In addition, several genes related to anthocyanidins were found on chromosomes 2A, 2B, 3B, 4A, 4B, and 6A, considering the MDC vs. TDL-2 comparison, while only three were detected on chromosomes 2B, and 6A for TDL-1 vs. TDL-2 (Supplementary Table S3).

Five transcript IDs localized on 6A chromosome resulted in disease resistance proteins and nucleotide-binding site and leucine-reach repeats proteins (NBS-LRR). Finally, powdery mildew resistance protein Pm3 was identified on chromosomes 1B and 7B (Table 3).

## Discussion

### Genetic Diversity in Tunisian Durum Germplasm

Durum wild and domesticated accessions are well adapted to extreme environments; therefore, they can represent a rich source of alleles useful to improve the elite cultivars with a greater tolerance to adverse climatic conditions, as well as to weeds, pests, and pathogens (De Vita and Taranto, 2019; Sansaloni et al., 2020; Kashif et al., 2021). In this challenging scenario, the exploration of the genetic diversity in indigenous durum landraces, using technological improvements in phenotypic and genotypic analysis, as well as the biotechnological and digital revolution, can be particularly effective (Taranto et al., 2018). Tunisian landraces preserve a rich reservoir of unexplored gene pools, which deserve to be recovered, characterized, and conserved (Slim et al., 2019; Robbana et al., 2021).

In this study, a panel of 43 durum wheat accessions, including lines selected within the most known Tunisian landraces and elite cultivars, was assessed using nine spike and kernel descriptors, and ~5,500 SNP markers with the aim to disclose the patterns of genetic diversity between landraces and cultivars. According to the IBS analysis, six accessions were discarded being considered synonymous accessions. This is not surprising because Tunisian durum landraces are transmitted by farmers from one generation to the next, with local names often linked to their historical origin or regional area or specific phenotypic traits, and they are often locally selected and exchanged (Robbana et al., 2019).

The genetic structure was performed using the 5,555 high-quality markers generated by the SNP genotyping. The two dimensions of PCoA clearly separated the modern cultivars from the Tunisian durum germplasm. These results were corroborated by the phylogenetic analysis, which was able to discriminate three major groups.

The first one (MDC) included the Tunisian cultivars, the international cultivars, and the lines selected within the most renowned traditional Tunisian landraces, namely, Mahmoudi (Mahmoudi glabre AP1), Bidi (Bidi-2), and Chili. The reason why some Tunisian cultivars are genetically and phenotypically very similar to the international cultivars could lie in the fact that some of them originate from the same progenitors. This is the case of Iride and Khiar cultivars that have Altar-84 as one of the progenitors, or Razzak that derives from Karim. MDC overlapped with cluster I generated by morphological data, except for Mahmoudi glabre AP1, Bidi-2, Maghrebi-72, and Wells that fall in cluster II or III, together with the other lines selected within landraces. The lines selected within Mahmoudi and Bidi landraces appear genetically related to one another, probably because both were introduced in Tunisia from Algeria. Mahmoudi is a landrace widely cultivated for its straw and grain yield, as well as its ability to produce a high yield under drought and heat stress conditions prevalent in southern Tunisia (Ayed and Slim-Amara, 2009; Ayed-Slama et al., 2018; Ouaja et al., 2021). Instead, Chili is an old cultivar introduced from France in the early 1930s with stable yield and a lower plant height; probably, these traits make Chili closer to modern cultivars than other Tunisian durum landraces.

The second group, named TDL-1, comprised the Tunisian pure lines characterized by parallel-sided spike, such as Derbessi AC1, Jenah Khotifa-2, Jenah Khotifa-3, Ward Bled, Sebei pubescent AP2, and Taganrog-2. This group also included the genotype Kahla, name by which the Tunisian farmers called the landrace Jenah Khotifa, characterized by a dark (black to purple) spike (Deghaïs et al., 2007).

The third group, named TDL-2, included the Tunisian lines characterized by fusiform spike shape and red glume color, except Jenah Khotifa-4.

The AMOVA results revealed that most of the variation was within groups. Indeed, accessions with the same name (i.e., Bidi, Jenah Khotifa) were different phenotypically and genotypically, although selected within populations. This suggests that originally the diversity within these populations was very wide, even greater than that among different landraces. This is in agreement with the results of Robbana et al. (2019, 2021), who observed a large variability within Bidi and Jenah Khotifa populations, using both phenotypic and molecular data.

The genetic indices revealed that the MDC cultivar group had a lower diversity than TDL landrace groups. This is expected as many studies indicate the loss in genetic diversity moving from landraces to the modern cultivars (Haudry et al., 2007; Laidò et al., 2013; Mangini et al., 2017; Mazzucotelli et al., 2020; Taranto et al., 2020). In addition, a moderate/high gene flow (Nm = 4.5) value was observed between the two Tunisian durum groups (TDL-1 vs. TDL-2). This can be explained by seed exchange between the local Tunisian farmers. Indeed, the seed exchange can allow the introgression of the alleles into the preexisting germplasm, determining gene flow (Ouaja et al., 2021).

The Bidi landrace, which was found both in the MDC (Bidi-2) and TDL-1 (Bidi-1) groups, deserves a separate comment. At the beginning of the 1900, Bidi landrace populations were widely multiplied and spread on Tunisian territory on a large scale (Bœuf, 1925). However, it is possible to recognize that Bidi has three different origins (Tunisia and Algeria and Morocco) (Bonjean and Angus, 2001). This can explain the fact that Bidi-1 and Bidi-2 belong to two different groups, namely, Bidi-2 (in the MDW group) may be a line selected from the Algerian Bidi 17, while Bidi-1 (in the TDL-1 group) may be a line selected by autochthonous landrace populations. The large variability of Bidi populations observed in this study is also in accordance with findings of Taranto et al. (2020) who analyzed several Bidi accessions derived from different gene banks. All Bidi accessions were different and clustered close to the most important Italian old cultivars, Cappelli and Margherito, suggesting that they were selected within Bidi populations.

### Candidate Genes in Divergent Regions Between Modern Varieties and Landraces

Based on genetic structure carried out using the nine descriptors and SNPs, it is possible to suggest that spike shape and density, as well as glume color, were the major key traits that drive the subdivision between Tunisian landraces and the durum cultivars, as recently suggested by Ouaja et al. (2021) and Robbana et al. (2021). To corroborate this observation, we investigated the divergent genomic regions between the three groups indicated by genetic analysis, with the aim to identify candidate genes responsible for genetic differentiation. The analysis confirmed that the Tunisian durum landraces (TDL-2) were highly divergent from MDC cultivars, particularly for genes involved in plant architecture, grain quality, and disease resistances.

The divergence analysis allowed us to identify a network of genes related to the regulation of flowering time, such as *FLOWERING LOCUS T* (*FT-B1*) located on chromosome 7B. Due to their impact on the phenology and flowering architecture of crops, *FT* genes have been a target of selection during domestication and adaption to new environments (Zheng et al., 2016). Recently, a *FT-B1*/*VRN-B3* (TraesCS7B02G013100) gene was identified in a quantitative trait locus (QTL) for spikelet number per spike in bread wheat (Brassac et al., 2021). This *FT-B1* gene, homologous to that found in this study on chromosome 7B (TRITD7Bv1G003900), was advised as the most likely candidate for the observed effect on spikelet number per spike, with a major effect on pre-flowering phases. In addition, we also found zinc finger CONSTANS transcription factors, which are involved in the mechanisms of regulation of *FT* locus (Zheng et al., 2022), confirming the pivotal role of the traits associated to flowering and plant architecture in the history of durum breeding programs. Other divergent genes such as transcription factors belonging to the superfamilies APETALA2/Ethylene-responsive element-binding proteins (*AP2/EREBPs*) and APETALA2/ethylene responsive factor (*AP2/ERF*) corroborate this observation. These transcription factors play a significant role in plant and spike architecture (Wu et al., 2011, 2012; Li et al., 2016). For example, the *PLANT ARCHITECTURE-RELATED GENE* (*PARG*), included in *AP2/EREBP* family, seems to regulate plant architecture-related and yield-related traits (Li et al., 2016). For *PARG* gene, two favorable haplotypes for *PARG-2A* were observed in modern cultivars with respect to landraces, implying that they were the object of breeding programs. Similar conclusions were found in the haplotypes analysis of *WHEAT FRIZZY PANICLE* (*WFZP*) gene, included in the *AP2/ERF* superfamily, that showed that the favorable alleles of *WFZP* associated with spikelet number per spike were preferentially selected during breeding (Li et al., 2021). In *Musa, Oryza, Prunus persica*, and *Capsicum, AP2/ERF* genes were also related to the domestication processes from wild ancestors or during breeding evolution (Zhang et al., 2012; Lakhwaniet et al., 2016; Sun et al., 2017; Jin et al., 2018).

In addition, the Tunisian durum landraces and durum cultivars showed a large phenotypic variation for spike density. Indeed, the three clusters comprised cultivars with spikes dense (cluster I), Tunisian lines with spike medium (cluster II), and Tunisian lines with spikes lax (cluster III). This morphological spike diversity is affected by genes related to spike architecture (i.e., FT-B1, AP2/EREBPs, AP2/ERF) and could be involved in response to high temperature. This suggested that the spike shape might be characteristic of certain Tunisian environmental conditions (lower arid vs. higher arid) as reported by Ouaja et al. (2021).

Divergent genes related to gluten composition and starch properties were found. This supported what was suggested by Boukid et al. (2018) who indicated that gluten protein and starch fractions play a role during the evolution of durum wheat from landraces to modern varieties in Tunisia. In addition, within the Tunisian germplasm some lines were characterized by reddish and purple glumes. Previous studies reported a genetic variation in kernel color between landraces and modern cultivars due to the action of genetic improvement on alleles and genes involved in peroxidase and polyphenol oxidase activity, carotenoid, and anthocyanin pathways (Ficco et al., 2014; Mangini et al., 2014; Taranto et al., 2015, 2020; Colasuonno et al., 2019; Ouaja et al., 2021). However, the anthocyanins pigments, as well as polyphenol oxidase, peroxidase, and carotenoid enzymes, are primarily important for plant adaptation under biotic and abiotic stress conditions (Jan et al., 2021; Naing and Kim, 2021; Šamec et al., 2021). In our study, we found several anthocyanidin and anthocyanin transcripts as divergent loci between Tunisian landraces and cultivars. It has been demonstrated that gene encoding for anthocyanidin reductase (Traes_2DL_A55995202) was induced in wheat by cereal cyst nematodes, probably increasing reactive oxygen species (ROS) scavenging and enhanced tolerance, as previously observed in tobacco (Luo et al., 2016; Qiao et al., 2019). We found that the orthologous gene (TRITD2Av1G265560) could be used as a candidate for future investigation in response to nematode. Recently, a study to understand the role and composition of the kernel headspace volatile organic compounds in response to stored grain pests highlighted the importance of characterizing and exploiting the pigmented wheat genotypes (with a high level of anthocyanins) in contrasting biotic stresses (Germinara et al., 2019).

Several divergent loci identified specific genomic regions involved in response to diseases such as rust (rust resistance-like protein RP1 G), powdery mildew (powdery mildew resistance *Pm*), or nucleotide-binding site and leucine-reach repeats proteins (NBS-LRR), such as the disease resistance proteins (RPM) and plant resistance gene analogs (*RGAs*), all belonging to the largest gene family of plant R genes coding for intracellular receptors that recognize the presence of pathogen (Shao et al., 2019). The *Pm3* genes were found in linkage with gliadin and glutenin genes on chromosome 1B, suggesting how the selection for resistant genotypes may have influenced the selection of modern cultivars with different gluten composition (De Santis et al., 2017; Visioli et al., 2021). Another class of divergent genes was that of *NAC* transcription factors, which form a large plant-specific gene family involved in the regulation of tissue development in response to biotic and abiotic stress (Puranik et al., 2012) by participating in the signal transduction pathways and the expression of downstream target genes (Zhang et al., 2018) in drought and salt resistance (Nguyen et al., 2019), and fungal and bacterial infections (Wang et al., 2007; Zhang et al., 2021). Our results suggest that Tunisian landraces can be considered a source of alleles for tolerance to biotic stresses. Confirming this hypothesis, Huhn et al. (2012) selected landraces moderately resistant to *Fusarium* head, while Ferjaoui et al. (2015), Ouaja et al. (2020), and Laribi et al. (2022) identified lines with resistance to *Septoria tritici* blotch, currently the most important foliar disease of durum wheat in Tunisia, where it causes an average yield loss of 5–35% (Fakhfakh et al., 2011).

The divergent genes identified between Tunisian landraces and modern cultivars belong to the same *T*. *turgidum* subspecies (*durum*). In our case, the divergent selection, acting in contrasting directions in two or more groups, may have arisen from the effect of selected breeding (Taranto et al., 2020). However, some of these divergent genes are well known as targets of the domestication syndrome, which is the process that led crops to evolve in a convergent way (Fullera et al., 2014; Woodhouse and Hufford, 2019). Examples of convergence genes (i.e., they shared functional orthology across species) underpinning favorable traits related to cereal domestication and adaptation processes were reported by Woodhouse and Hufford (2019). Our results were consistent with what was indicated by Woodhouse and Hufford (2019), revealing that we found genes, such as *FT* and vernalization (*VRN3*), involved in convergent selection among rice, wheat, sunflower, and barley (Lenser and Theißen, 2013; Wang et al., 2013). Starch genes were also recognized to be involved in convergent selection in crop species such as rice, wheat, maize, foxtail millet, barley, sorghum, and maize millet (Li et al., 2012). Finally, *LEA* genes associated with cold tolerance have undergone a convergent selection between barley and wheat (Artur and Kajala, 2021). The study of causal loci underlying important traits such as domestication and adaptation through the comparative analyses of cereals and their wild relatives will play an important role in clarifying the timing of selection and identifying useful alleles to enhance and assist modern breeding.

In conclusion, our results confirm that Tunisian durum landraces carry useful alleles for diversifying and widening the genetic basis for durum breeding for durable resistance, which is the key for the sustainability of durum wheat. Hence, the need to ensure a long-term conservation of Tunisian durum wheat landraces is mandatory in the light of the ongoing genetic erosion.

## Data Availability Statement

The filtered VCF data presented in the study are deposited in the Mendeley Data repository, doi: https://doi.org/10.17632/9hnjw77mf2.2.

## Author Contributions

GM: supervision. GM and FT: conceptualization. MM, FT, and GM: methodology and data curation. MM, EB, and FT: formal analysis. MM, EB, and GM: investigation. PD, RC, and CM: resources. MM and EB: writing—original draft. PD, FT, and GM: writing—review and editing. All authors reviewed and approved the final version of this manuscript.

## Funding

This research was supported by the Ministry of Agricultural, Food and Forestry Policies (MIPAAF) with the project RGV-FAO and the Italian Cooperation to the Tunisian Ministry of Local Affairs and Environment with the project Ressources phytogénétiques tunisiennes mieux conservées et valorisées.

## Conflict of Interest

CM was employed by Spin Off Sinagri s.r.l. The remaining authors declare that the research was conducted in the absence of any commercial or financial relationships that could be construed as a potential conflict of interest.

## Publisher's Note

All claims expressed in this article are solely those of the authors and do not necessarily represent those of their affiliated organizations, or those of the publisher, the editors and the reviewers. Any product that may be evaluated in this article, or claim that may be made by its manufacturer, is not guaranteed or endorsed by the publisher.
