# Supplementary material for: Comparative Genetic Analysis of Durum Wheat Landraces and Cultivars Widespread in Tunisia
Source: Front Plant Sci. 2022 Jul 13;13:939609. doi: 10.3389/fpls.2022.939609 (PMC9326505; doi:10.3389/fpls.2022.939609)
Supplement: Supplementary file 1 [file Presentation_1.PPTX]

## Slide 1
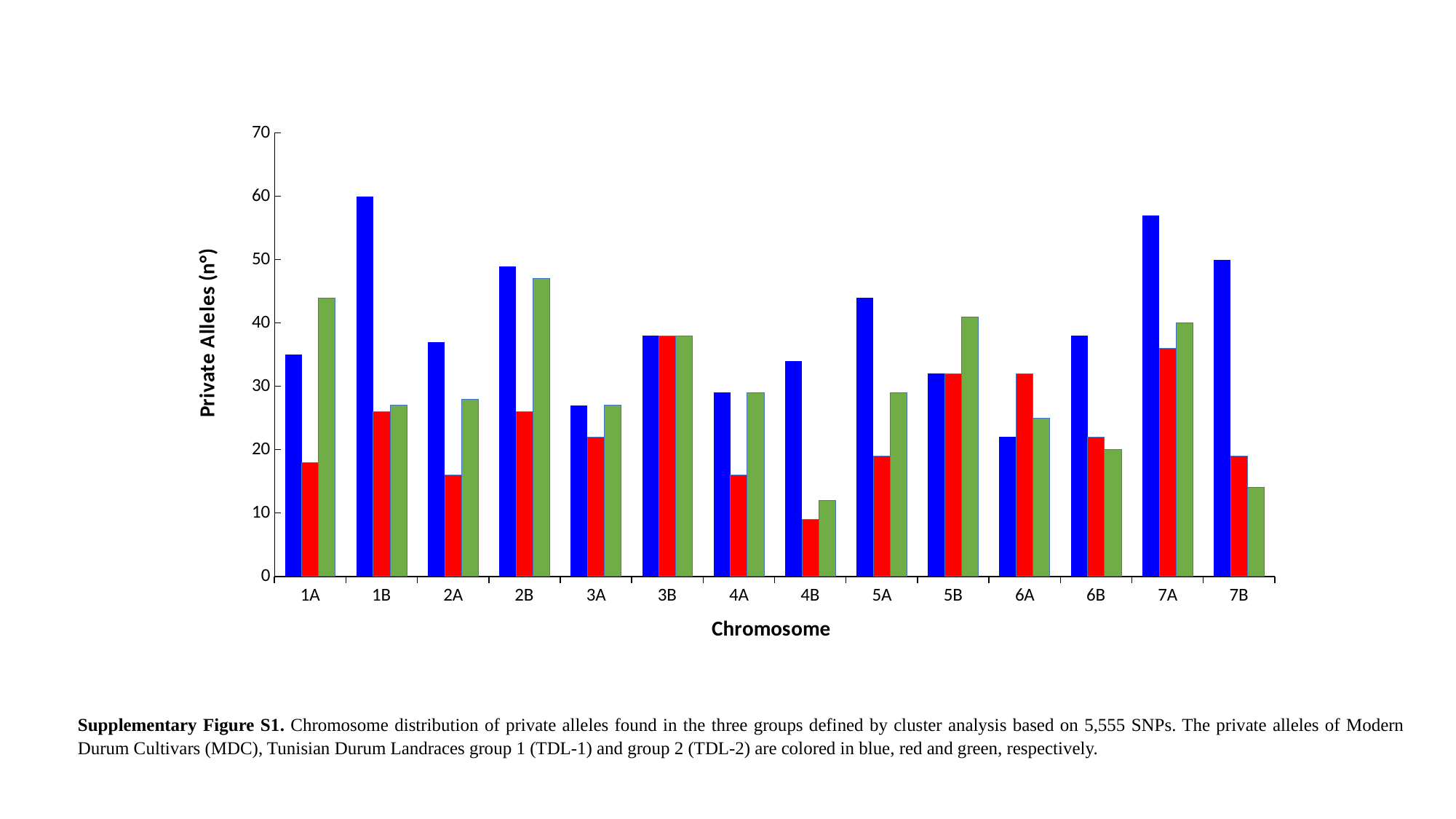

### Chart
| Category | | | |
|---|---|---|---|
| 1A | 35.0 | 18.0 | 44.0 |
| 1B | 60.0 | 26.0 | 27.0 |
| 2A | 37.0 | 16.0 | 28.0 |
| 2B | 49.0 | 26.0 | 47.0 |
| 3A | 27.0 | 22.0 | 27.0 |
| 3B | 38.0 | 38.0 | 38.0 |
| 4A | 29.0 | 16.0 | 29.0 |
| 4B | 34.0 | 9.0 | 12.0 |
| 5A | 44.0 | 19.0 | 29.0 |
| 5B | 32.0 | 32.0 | 41.0 |
| 6A | 22.0 | 32.0 | 25.0 |
| 6B | 38.0 | 22.0 | 20.0 |
| 7A | 57.0 | 36.0 | 40.0 |
| 7B | 50.0 | 19.0 | 14.0 |Supplementary Figure S1. Chromosome distribution of private alleles found in the three groups defined by cluster analysis based on 5,555 SNPs. The private alleles of Modern Durum Cultivars (MDC), Tunisian Durum Landraces group 1 (TDL-1) and group 2 (TDL-2) are colored in blue, red and green, respectively.

## Slide 2
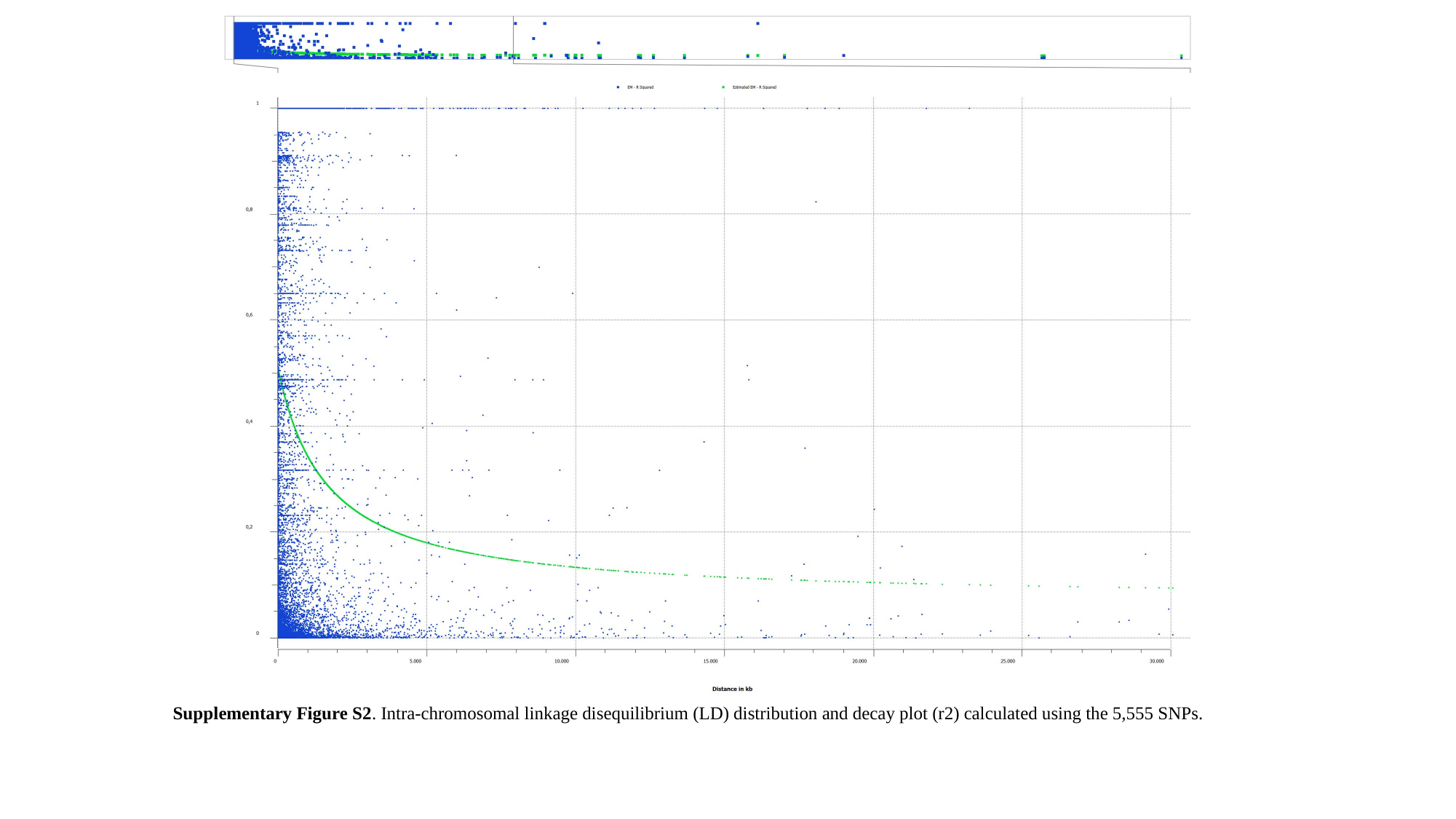

Supplementary Figure S2. Intra-chromosomal linkage disequilibrium (LD) distribution and decay plot (r2) calculated using the 5,555 SNPs.
